# *Tamarindus indica* Shells Powder Enhances Growth Performance, Hemato-Biochemical Parameters, Nutrient Utilization, and Gut Health in Broiler Chickens

**DOI:** 10.3390/vetsci13060566

**Published:** 2026-06-08

**Authors:** Thanyarat Somsu, Wandee Udomuksorn, Kasemsiri Chandarajoti, Sathianpong Phoopha, Jiraporn Khanansuk, Suthinee Sangkanu, Chatchai Wattanapiromsakul, Michael Wink, Sukanya Dej-adisai

**Affiliations:** 1 Department of Pharmacognosy and Pharmaceutical Botany, Faculty of Pharmaceutical Sciences, Prince of Songkla University, Hat Yai 90112, Songkhla, Thailand; thanyarat.s@rmutsv.ac.th (T.S.); jiraporn.kha@psu.ac.th (J.K.); suthinee.s@psu.ac.th (S.S.); chatchai.w@psu.ac.th (C.W.); 2 Division of Health and Applied Science, Faculty of Science, Prince of Songkla University, Hat Yai 90112, Songkhla, Thailand; wandee.u@psu.ac.th; 3 Department of Pharmaceutical Chemistry, Faculty of Pharmaceutical Sciences, Prince of Songkla University, Hat Yai 90112, Songkhla, Thailand; kasemsiri.c@psu.ac.th; 4 Traditional Thai Medical Research and Innovation Center, Faculty of Traditional Thai Medicine, Prince of Songkla University, Hat Yai 90112, Songkhla, Thailand; sathianpong.p@psu.ac.th; 5 Institute of Pharmacy and Molecular Biotechnology, Heidelberg University, 69120 Heidelberg, Germany; wink@uni-heidelberg.de

**Keywords:** tamarind shell powder, broiler chickens, diet supplementation, gut leakage analysis, hematological parameters

## Abstract

Broiler production has frequently faced economic losses due to infectious diseases caused by pathogenic microorganisms. These problems are commonly resolved using antibiotics, but doing so could lead to antibiotic resistance and impair food safety. This study evaluated the effects of a tamarind (*Tamarindus indica* L.) shell powder (TSP) supplementation on the growth performance and overall health status in broiler chickens. A total of 375 one-day-old male Ross 308 broilers were randomly assigned to five dietary treatment groups in a completely randomized design. Broilers received either a basal diet; antibiotic-supplemented diet; or diet supplemented with TSP at 1 × MIC, 16 × MIC, or 32 × MIC daily for 42 days. The results showed that treatment 4 (TSP 16 × MIC—64 mg per bird) had the best growth performance in broilers. Intestinal permeability measurements assessed using 4 kDa fluorescein isothiocyanate-conjugated dextran (FITC–dextran) showed that broilers fed the treatment 5 (TSP 32 × MIC—128 mg per bird) diet had significantly lower FITC–dextran concentrations in all intestinal segments (*p* < 0.05) and were determined to have higher serum FITC–dextran than the control group. The hematological parameters can significantly reduce serum cholesterol and triglycerides. Finally, the application of tamarind shell powder promoted probiotic proliferation within the broilers’ gastrointestinal tract and mitigated *Enterobacteriaceae* infections, demonstrating comparable efficacy to the antibiotic-treated control group. This research suggests that tamarind shell powder supplementation, especially at a medium dosage (treatment 4), may beneficially influence gut morphology, modulate the gut microbiota, and enhance intestinal health in broiler chickens.

## Simple Summary

This study demonstrates that supplementation of a broiler diet with tamarind shell powder (TSP) can positively influence the growth performance and gut health of broiler chickens. The findings indicate that TSP supplementation enhances feed intake, weight gain, and average daily gain, while also reducing mortality rates. Additionally, TSP contributes to improved intestinal integrity and reduced inflammation. The supplementation also beneficially modulates serum lipid profiles without adversely affecting hematological parameters. Overall, TSP, especially at a medium dose, shows promise as a natural feed additive to promote growth and gastrointestinal health in broiler chickens.

## 1. Introduction

Broiler meat is an important protein source in many countries and is becoming popular all over the world due to its good digestibility. To provide people adequately with this protein source, broiler chickens have to be bred in large quantities [1]. However, infectious diseases caused by pathogenic bacteria have resulted in economic losses on broiler farms. Antibiotics must be used to solve these issues, but they may cause antibiotic resistance and risk food safety [2]. In addition, farmers use antibiotics for growth promotion in livestock, even though this practice is strongly prohibited globally [3]. In addition to the detrimental effects of antibiotic accumulation in meat, it has been discovered that 75–90% of antibiotics are not metabolized in the digestive tract of animals and are eventually released into the environment from livestock wastewater treatment systems, which may further contribute to the emergence of antibiotic-resistant bacteria in the environment [4]. This growing global concern has led to a complete ban or restricted use of antibiotic growth promoters (AGPs), and poultry producers have faced increasing challenges in maintaining bird health and productivity without relying on conventional antibiotics [5]. Consequently, research is needed for alternatives to antibiotics. Such an alternative would be a focus on natural products that can enhance growth and improve the health of broiler chickens. Natural products or secondary plant metabolites can be obtained in different ways, including isolated compounds, plant extracts, essential oils, herbs, and even agricultural by-products. Plants are rich in bioactive compounds such as phenolics, flavonoids, terpenoids, and organic acids, which exhibit antimicrobial, antioxidant, and anti-inflammatory activities [6,7,8]. Currently, several research studies have attempted to incorporate agricultural waste or by-products into broiler diets to reduce feeding costs, especially if these products can inhibit pathogenic bacteria and reactive oxygen species, enhance growth performance, and improve gut health, making them promising alternatives to conventional antibiotics.

Tamarind (*Tamarindus indica* L.) is a widely cultivated legume (Fabaceae) in tropical regions. The evergreen tree produces oblong, greyish-brown indehiscent pods with up to 12 seeds; they are surrounded by a fleshy edible fruit pulp. The pulp, which is a mild laxative, is commonly used in its crude or purified form. The shells of the remaining pods are a by-product of pulp production [7]. Normally, tamarind shells are considered an agricultural by-product; however, they contain various bioactive compounds, including flavonoids, tannins, and terpenoids [9]. These phytochemical compounds have been reported to exhibit antioxidant, antimicrobial, and anti-inflammatory activities, which are relevant to intestinal health and physiological function in livestock [10]. Many studies demonstrated the biological activities of tamarind-derived compounds; however, in vivo studies evaluating the application of tamarind shell-derived products in broiler nutrition are still limited [10,11,12,13,14,15]. In particular, there is still insufficient evidence regarding their effect on growth performance, intestinal morphology, and gut microbial balance. Furthermore, important knowledge gaps remain regarding dose-dependent response, regulation of intestinal barrier function, and modulation of gut microbiota [12,14,15]. Based on the previous studies about the bioactive properties of *Tamarindus indica* and the limited in vivo evidence in broiler chickens, it was hypothesized that dietary supplementation with tamarind shell powder may positively modulate growth performance and health status in broiler chickens.

Therefore, this study explores the effect of the supplementation of broiler diet with tamarind shells on growth performance, hemato-chemical, and histological parameters, and pathogenic and lactic acid bacterial population of broiler chickens. In addition, the improvement of probiotic growth and the reduction of the pathogens in broilers by the tamarind extracts were investigated.

## 2. Materials and Methods

### 2.1. Animal Ethical Statement

All animal experiments were conducted according to the national guidelines and were approved by the Institutional Animal Care and Use Committee of Prince of Songkla University (Hat Yai, Songkhla, Thailand). The number of animal ethics document was MHESI 68014/1038.

### 2.2. Tamarind Shell Powder (TSP) Preparation

For this project, we collected about 100 kg sour giant tamarind fruits (*Tamarindus indica* L.), a fruit commonly found in markets in Thailand. The tamarind pods were peeled, and the pulp and seeds were removed. Then the shells were washed and dried at 50 °C for 72 h or until completely dry. Subsequently, the dried tamarind shells were ground into small particles measuring about 149 µm.

### 2.3. Tamarind Shell Extraction

Tamarind shell powder was subjected to maceration with 80% ethanol at room temperature for three days, followed by filtration through coarse filter paper. The resulting residue was then repeatedly extracted by re-macerating it at room temperature. The filtrations obtained from each extraction were concentrated under reduced pressure using a rotary evaporator set at 45 °C and subsequently pooled. The final crude extracts were stored at 4 °C until further analysis.

### 2.4. Antibacterial Activity Determination

#### 2.4.1. Preparation of Bacterial Strains

The experimental procedure involved the utilization of bacterial strains, including *Salmonella typhimurium*, *Salmonella enteritidis*, *Escherichia coli*, and *Klebsiella pneumoniae*. Each strain was initially cultured in Trypticase Soy Broth (TSB) and incubated at 37 °C for 24 h. Subsequently, aliquots from the TSB cultures were streaked onto Trypticase Soy Agar (TSA) plates and incubated under the same conditions for 24 h. Pure colonies obtained from the TSA plates were then transferred to TSB for approximately 4 h at 37 °C. The bacterial suspensions were adjusted to an optical density at 625 nm (OD_625_) within the range of 0.08 to 0.13, corresponding to a 0.5 McFarland standard (~1.5 × 10^8^ CFU/mL). Prior to conducting broth microdilution assays, the pathogenic bacterial suspensions were further diluted at a 1:100 ratio with sterile 0.9% saline solution to obtain 10^6^ CFU/mL.

#### 2.4.2. Minimal Inhibitory Concentration (MIC) and Minimal Bactericidal Concentration (MBC) Determination

The procedures employed to detect the Minimum Inhibitory Concentration (MIC) and Minimum Bactericidal Concentration (MBC) conformed to the protocols outlined by the Clinical and Laboratory Standards Institute (CLSI) [16], with certain modifications. Ten serial two-fold dilutions of tamarind crude extract were prepared in Tryptic Soy Broth (TSB), with concentrations from 512 mg/mL to 1 mg/mL. Subsequently, 50 μL of each diluted extract was dispensed into individual wells in triplicate. To each well, 50 μL of bacterial inoculum standardized to 10^6^ CFU/mL was added, resulting in final extract concentrations ranging from 256 mg/mL to 0.5 mg/mL. The plates containing all bacterial strains were incubated at 37 °C for 15 h. Following incubation, 30 μL of a 0.1% resazurin solution was added to each well, with an additional incubation of 3 h to allow for colorimetric assessment, as adapted from previous protocols [17]. A color change from purple to pink indicated bacterial growth (negative result), whereas maintenance of a blue or purple hue signified growth suppression (positive result). The antibiotic enrofloxacin served as positive control, given its widespread use in poultry for treating respiratory, renal, and digestive infections while uninoculated and inoculated mediums without treatment served as sterility and bacterial growth controls, respectively. All assays were performed in triplicate biological experiments with technical triplicates to ensure reproducibility. The MIC was determined as the lowest extract concentration that resulted in no color change (retention of blue color). To establish the MBC, aliquots from wells exhibiting no visible growth were sub-cultured onto Tryptic Soy Agar (TSA) plates and incubated under appropriate conditions. The MBC was defined as the lowest concentration at which no bacterial growth was observed upon sub-culturing. All experiments were conducted in triplicate.

### 2.5. Experimental Animal Study Design, Housing, and Environmental Management of Broilers

A total of 375 one-day-old male Ross 308 broiler chickens (average body weight, 50.69 ± 0.01) were purchased from a local commercial hatchery and transferred to the experimental unit of Division of Animal Sciences, Nakhon Si Thammarat, Thailand. All broilers were reared under an intensive production system in a closed-house facility with strict biosecurity measures implemented throughout the experimental period. The experiment consisted of five experimental groups, each of which had 75 broiler chickens divided into 3 floor-pen replicates (25 broiler chickens per pen). Each floor pen measured 1 × 5 × 1 m (width × length × height) and was separated with partitions to prevent interaction among replicates. Broiler chickens were housed at a stocking density of 5 birds/m^2^ on rice husk litter bedding approximately 5 cm in thickness, over the 42-day rearing period. Feed and drinking water were provided ad libitum, and broiler chickens had continuous access to them (24 h/day) throughout the study. Management practices, including lighting schedules, vaccination protocols, and routine husbandry procedures, were implemented in accordance with the guidelines outlined in the Feeding Management Regulations for broilers [18,19,20,21].

The experiments were conducted in four distinct phases: days 1–10, 11–24, 25–39, and 40–42. The dietary formulations adhered to the nutritional requirements specified for broilers [17]. Detailed compositions and nutrient levels of the diets are provided in Table 1. Each phase was assigned to five experimental groups, each comprising three replicates of 25 birds. Treatment 1 (control) was provided with a basal diet without any supplementation. The remaining groups received the basal diet supplemented as follows: treatment 2 (the antibiotic enrofloxacin 10 mg/kg) with enrofloxacin included in the diet; treatment 3 (TSP 1 × MIC) with tamarind shell powder at a concentration of 1 × MIC (4 mg per bird); treatment 4 (TSP 16 × MIC) with tamarind shell powder at 16 × MIC (64 mg per bird); and treatment 5 (TSP 32 × MIC) with tamarind shell powder at 32 × MIC (128 mg per bird). The experimental diets were provided in ground form throughout the experimental period.

### 2.6. Health Monitoring and Welfare Management

All birds followed a standard commercial vaccination program for Ross 308 broilers. Vaccination started at the hatchery with a combined infectious bronchitis and Newcastle disease vaccine delivered via a coarse spray. A later vaccination used a combined infectious bronchitis (H120 strain) and Newcastle disease vaccine (LaSota strain) on day 7 and 14, and infectious bursal disease vaccine (Winterfield 2512 strain) on day 10 and 18 via drinking water. Birds’ health was monitored twice daily for animal behavior, general condition, and clinical signs. Mortality and euthanasia cases were recorded daily. The criteria for euthanasia included inability to access feed and water, severe injury, or systemic infection [19].

### 2.7. Growth Performance Measurement

Body weight and feed consumption were assessed weekly using randomly selected from each treatment group (3 broilers/replicate; 3 replicates/treatment). Growth performance measurements were conducted in the morning, before feed administration, to reduce variation associated with gastrointestinal content. The pen was considered the experimental unit for all growth performance analyses [19,22]. Growth performance parameters, including body weight gain (BWG), average daily feed intake (ADFI), average daily gain (ADG), and average feed conversion ratio (AFCR), were computed for two distinct phases: days 1–21 and days 22–42. Additionally, mortality rates were documented and analyzed [19,22].

### 2.8. Hematological and Serum Biochemical Measurement

On days 21 and 42, blood samples were obtained from 9 broilers/treatment via wing-vein puncture. The hematological parameters assessed included packed cell volume (PCV); total red blood cell (RBC) count; total white blood cell (WBC) count; and differential leukocyte counts, encompassing lymphocytes, monocytes, heterophils, eosinophils, and basophils. Serum biochemical analyses encompassed measurements of alanine transaminase (ALT), creatinine, total protein, albumin, globulin, cholesterol, triglycerides, albumin-to-globulin (A:G) ratio, and alkaline phosphatase (ALP) [22,23,24].

### 2.9. Gut Leakage Measurement

Gut leakage assessment was conducted to evaluate enteric inflammation and mucosal permeability. Blood samples were collected via wing-vein puncture and intestinal tissues were collected from 9 randomly selected birds per group. The birds were fasted for 2 h before administrated oral fluorescein isothiocyanate–dextran (FITC–dextran). After 2.5 h, blood was collected from the wing vein to measure the level of serum FITC–dextran. All sampled broilers were continuously fasted for 1 h and then were euthanized, and tissue samples such as duodenum, jejunum, ileum, and cecum were collected. Approximately 2 cm long segments of intestinal tissues were flushed with sterile phosphate-buffered saline (PBS).

Serum samples were subjected to centrifugation at 1000× *g* for 15 min at 4 °C to facilitate separation. The resulting supernatants were diluted in PBS at a 1:1 ratio. Fluorescence intensity was measured using a Varioskan^TM^ LUX Multimode microplate reader (Thermo-Fisher Scientific, Waltham, MA, USA) at excitation and emission wavelengths of 485 nm and 528 nm, respectively, to quantify FITC–dextran levels. The fluorescence data obtained from the samples were subsequently converted to micrograms of FITC–dextran per milliliter of serum by referencing a standard curve generated from known concentrations of FITC–dextran.

All intestinal tissues were carefully washed with Hanks’ Balanced Salt Solution (HBSS), and excess fluid was removed. The tissues were then weighed and placed into vials containing 10 mL of HBSS supplemented with glutamine and antimicrobial agents. The vials were incubated at 42 °C for a duration of 2 h to facilitate the release of FITC–dextran into the buffer from the tissue samples. Fluorescence intensity of the released FITC–dextran was measured at an excitation wavelength of 485 nm and an emission wavelength of 528 nm using a Varioskan^TM^ LUX Multimode microplate reader (Thermo-Fisher Scientific, Waltham, MA, USA). The fluorescence data obtained were subsequently converted to concentration values expressed in micrograms per milliliter of tissue, based on a standard curve generated from known FITC–dextran concentrations [22,25,26,27]. The FITC–dextran standard curve demonstrated linearity (R^2^) 0.999. The intra-assay coefficient of variation (CV) was below 10%, indicating acceptable analytical precision and assay repeatability [25,26,27].

### 2.10. Determination of Intestine Bacterial Population

Samples were obtained by cloacal swabs stored in transport medium tubes and maintained at 4 °C prior to analysis. Bacterial isolation was conducted in accordance with ISO 6579:2001/AMD 1:2007 [28] and BAM guidelines [29], involving streaking onto Tryptic Soy Agar (TSA) plates, followed by incubation at 37 °C for 24 h. Colonies exhibiting white, greasy circular characteristics were subjected to further testing using selective media, namely Xylose Lysine Deoxycholate (XLD) agar and Eosin–Methylene Blue (EMB) agar, with incubation at 37 °C for 24 h. Confirmatory identification was achieved through biochemical testing, including Motility–Indole–Lysine (MIL) medium, Lysine Iron Agar (LIA), Triple Sugar Iron (TSI) agar, and Simmons Citrate agar, all incubated at 37 °C for 24 h [28,29].

Intestinal tissue samples were collected from the duodenum, jejunum, ileum, cecum, and colon at 21 and 42 days of age. These samples (10 g) were preserved in 30 mL of phosphate-buffered saline (PBS, pH 7.4), within sterile containers and stored at 4 °C prior to analysis. The initial step involved immersing the samples in 70% ethanol for 30 s, repeated twice, followed by rinsing with sterile water to ensure surface sterilization. Subsequently, each sample was diluted with 30 mL of sterile 0.9% normal saline and incubated at 37 °C for 24 h under anaerobic conditions to promote bacterial growth. The third step entailed quantifying lactic acid bacteria (LAB) colonies that were serially diluted (10^−1^ to 10^−8^), and using the drop plate method on de Man, Rogosa, and Sharpe (MRS) agar supplemented with 0.02% bromocresol purple, followed by incubation at 37 °C for 48 h. Colony-forming unit (CFU) were calculated and expressed as log CFU/g of intestinal content. Finally, isolated colonies were subjected to Gram staining; catalase testing; and additional probiotic characteristic assessments, such as low pH (acid) tolerance, bile salt tolerance, gastric juice tolerance, intestinal fluid tolerance, protein digestion, starch digestion, and fat digestion [30,31,32].

### 2.11. Antimicrobial Activity of Lactic Acid Bacteria

The antimicrobial activity was evaluated by the cross-streak technique. Each isolate of LAB was streaked on TSA in a straight line and incubated at 37 °C for 48 h. After incubation, the plates were seeded with pathogenic organisms via a single streak at a 90° angle of LAB isolates and incubated at 37 °C for 24 h. The antimicrobial activity of LAB strains on pathogenic bacteria was analyzed by measuring the size of the inhibitory zone. The antagonist percentage of LAB strains to pathogenic bacteria was estimated [33].

### 2.12. Bacterial Strain Identification

The representative isolates of pathogenic bacteria were selected for molecular identification based on phenotypic appearance and frequency of occurrence among isolates found in broiler chickens. For LAB, representative isolates were selected based on colony morphological characteristics and functional screening results. The isolates that showed the highest potential based on the LAB functional test were selected to reflect the dominant functional and phenotypic traits within the LAB group.

The bacterial identification was performed using 16S rDNA sequencing, and all samples were analyzed by the Thailand Bioresource Research Center (TBRC). Briefly, the DNA templates for PCR amplification were prepared by using a Genomic DNA mini kit (blood/culture cell). DNA coding for 16S rDNA regions was amplified by means of PCR with Taq polymerase. A PCR product for sequencing 16S rDNA regions was prepared by using the following two primers, 20F (5′-GAG TTT GAT CCT GGC TCA G-3′, positions 9–27 on 16S rDNA; and 1500R (5′-GTT ACC TTG TTA CGA CTT-3′), position 1509–1492 on 16S rDNA by the *E. coli* numbering system. The PCR amplification was carried out with DNA Engine Dyad^®^ Thermal Cycle (Bio-Rad Laboratories, Hercules, CA, USA). A 100 μL reaction mixture was used containing 15–20 ng of DNA template; 2.0 μmoles each of the two primers; 2.5 units of Taq polymerase; 2.0 mM of MgCl_2_; 0.2 mM of dNTP; and 10 μL of 10 ×Taq buffer, pH 8.8, containing (NH_4_)_2_SO_4_, which comprised 750 mM of Tris-HCl, 200 mM of (NH_4_)_2_SO_4_, and 0.1% of Tween 20. The PCR amplification was programmed to carry out an initial denaturation step at 94 °C for 1 min, annealing at 50 °C for 1 min, and elongation at 72 °C for 2 min, followed by a final amplification step at 72 °C for 3 min. The PCR product was analyzed by 0.8% (*w*/*v*) agarose gel electrophoresis and purified with a GeneHlow^TM^ Gel/PCR Kit [34,35,36,37,38].

The sequencing of the purified PCR products was performed on an ABI Prism^®^ 3730XL DNA (Applied Biosystems, Foster City, CA, USA) Sequence by sequencing service provider. The four primers 27F (5′-AGA GTT TGA TCM TGG CTC AG-3′), 800R (5′-TAC CAG GGT ATC TAA TCC-3′), 518F (5′-CCA GCAGCC GCG GTA ATA CG-3′), and 1492R (5′-TAC GGY TAC CTT GTT ACG ACT T-3′) for double-strand 16S rDNA sequencing were used [34].

### 2.13. Phylogenetic Analysis

The nucleotide sequences obtained from all primers were assembled using Cap contig assembly program, an accessory application in BioEdit (Biological sequence alignment editor) Program “https://bioedit.software.informer.com/7.2/ (accessed on 15 May 2025)”. The identification of phylogenetic neighbors was initially carried out using BLAST+ version 2.17.0 against GenBank (Nucleotide BLAST at NCBI) against 16S rDNA sequence database of validly published prokaryote. The sequences with the highest scores were calculated via pairwise sequence similarity using global alignment algorithm [35]. The phylogenetic tree was constructed with the neighbor-joining methods in MEGA 12 program. The neighbor-joining algorithm was calculated with Kimura two-parameter model. Bootstrap analysis was used to evaluate the topology of tree based on 1000 replications.

### 2.14. Intestinal Morphology and Nutrient Utilization

On day 42, broiler chickens were randomly selected and subjected to a fasting period of two hours prior to necropsy. Segments approximately 2 cm in length from the intestinal tissues—including the duodenum, jejunum, ileum, cecum, and colon—were dissected from each group and fixed in a 10% neutral buffered formalin solution. The intestinal tissues were dehydrated through a series of ethanol solutions (70%, 90%, 96%, and 100%), followed by clearing in xylene and embedding in paraffin. The resulting paraffin blocks were sectioned using a microtome to obtain four sections per broiler sample, each 5 μm thick, with sections spaced intermittently. These sections were stained with hematoxylin and eosin (H&E) and examined under a light microscope. Morphometric measurements included villus height (or length), villus width, and crypt depth. Subsequently, the villous absorptive surface area was calculated using the appropriate formula [39,40,41]:

(1) $$\mathrm{villus}\;\mathrm{absorptive}\;\mathrm{surface}\;\mathrm{area}\;({\mathrm{mm}}^{2})\;=\;2\pi \;\times \;\frac{\left[\mathrm{Villus}\;\mathrm{width}\right]}{2}\;\times \;[\mathrm{Villus}\;\mathrm{height}]$$

### 2.15. Statistical Analysis

The sample size was calculated using G*Power software (version 3.1.9.7), assuming an effect size (f) of 0.25, α = 0.05, and power (1-β) 0.80. The data are presented as the mean ± standard error (S.E.) and were analyzed using the IBM SPSS Statistics software package (version 25.0; IBM Corporation, Chicago, IL, USA). All data, including growth performance parameters, hematological parameters, gut leakage indicators, nutrient utilization parameters, and bacterial investigation of gastrointestinal tract, were initially assessed for normality of distribution using the Shapiro–Wilk test and for homogeneity of variance using Levene’s test. The pen was defined as the experimental unit for all measured parameters. One-way analysis of variance (ANOVA) was employed to assess differences among the experimental groups, and Duncan’s multiple range test (DMRT) was conducted for post hoc multiple comparisons. Statistical significance was established at a threshold of *p* < 0.05 [19].

## 3. Results

### 3.1. Antibacterial Activity

This investigation demonstrated that extracts derived from tamarind shell powder possessed broad-spectrum antibacterial properties, effectively inhibiting all three pathogenic bacterial strains tested at a minimum inhibitory concentration (MIC) of 4 mg/mL. The minimum bactericidal concentration (MBC) was determined to be 8 mg/mL for *E. coli*, and 64 mg/mL for *S. typhimurium* and *S. enteritidis*. As expected, the antibiotic enrofloxacin was more powerful and exhibited significant antibacterial activity at concentrations below MIC and MBC values of tamarind shells extracts (Table 2).

### 3.2. Growth Performance of Broiler Chickens

Broiler chickens were treated with a diet supplemented with tamarind shell powder. The growth performance results are presented in Table 3. During the first period (1–21 days), treatment 4 (TSP 16 × MIC) exhibited significantly higher values of ADFI, BWG, and ADG compared to the other treatments. On the other hand, the AFCR of treatment 4 (TSP 16 × MIC) was a relatively higher value compared with treatment 1 (control group). During the second period (22–42 days), treatment 3 (TSP 1 × MIC) exhibited a higher value of BWG and ADG, whereas treatment 4 (TSP 16 × MIC) showed a significantly higher value of ADFI. Overall growth performance (1–42 days), treatment 4 (TSP 16 × MIC) exhibited a significantly higher value of ADFI compared with treatment 1 (control group). Significant differences were observed in AFCR among treatment groups. Additionally, the administration of treatments 4 (TSP 16 × MIC) and 5 (TSP 32 × MIC) of tamarind shell powder resulted in a statistically significant reduction in mortality rates (*p* < 0.05) relative to the control group (treatment 1).

### 3.3. Hematological and Serum Biochemical

As presented in Table 4, the hematological parameters of chickens assessed on day 21 demonstrated significant differences through experimental groups. Specifically, treatments 2 and 5 exhibited notably elevated PCV values. Treatment 3 (TSP 1 × MIC) was associated with a significantly increased RBC count. Additionally, WBC counts were markedly higher in treatments 4 (TSP 16 × MIC) and 5 (TSP 32 × MIC). On day 21, differential leukocyte profiles exhibited increased lymphocyte percentages and decreased monocyte and heterophil percentages in TSP-supplemented groups compared with the control group (*p* < 0.05). Eosinophil percentages were elevated in treatments 2 (enrofloxacin) and 5 (TSP 32 × MIC). On day 42, dietary treatments significantly affected monocyte, eosinophil, and basophil percentages, whereas lymphocyte and heterophil percentages were not significantly different among treatment groups. Furthermore, treatments 4 and 5 continued to display significant alterations in PCV on day 42.

As presented in Table 5, the biochemical serum parameters of broiler chickens determined on day 21 indicated a significant elevation in the albumin-to-globulin (A:G) ratio, whereas the levels of ALP and globulin remained statistically unchanged. Additionally, no significant differences were observed in ALT level or total protein levels among the experimental groups. Serum cholesterol and triglycerides were significantly decreased in TSP-supplemented groups. On day 42, ALT levels were significantly lower in treatments 3 (TSP 1 × MIC) and 4 (TSP 16 × MIC) compared with the control group. The creatinine levels were significantly lower in treatments 4 (TSP 16 × MIC) and 5 (TSP 32 × MIC) compared with the control group.

### 3.4. Gut Leakage Analysis in Broiler Chickens

The effects of TSP supplementation on intestinal permeability were evaluated by quantifying serum fluorescein isothiocyanate–dextran (FITC–dextran) levels for a permeability marker, as detailed in Table 6. Results indicated that broiler chickens supplemented with tamarind shells powder exhibited slightly increased serum FITC–dextran concentrations relative to the control group (treatment 1). Notably, the groups receiving the medium (treatment 4, TSP 16 × MIC) and high dose of TSP (treatment 5, TSP 32 × MIC) demonstrated a statistically significant elevation in serum FITC–dextran levels compared to the control group. The intestinal tissue-associated FITC–dextran concentrations varied among intestinal segment and treatment groups. In the duodenum, treatment 4 (TSP 16 × MIC) showed significantly increased FITC–dextran compared with treatments 2 (enrofloxacin) and 5 (TSP 32 × MIC), while the lowest value was observed in treatment 5 (TSP 32 × MIC). In the jejunum and cecum, no significant differences were observed among treatment groups. And in the ileum, treatment 3 (TSP 1 × MIC) showed significantly decreased FITC–dextran compared with the control group and other treatment groups.

### 3.5. Intestinal Morphology and Nutrient Utilization in Broiler Chickens

**Figure 1:**
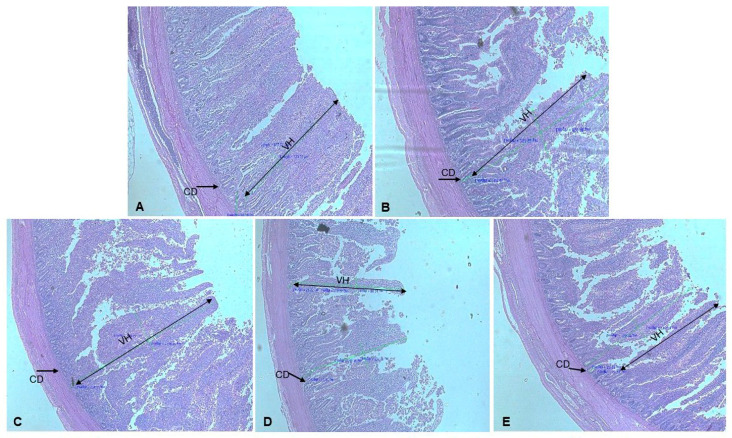
Histopathological sections of duodenum in broiler chickens stained with hematoxylin and eosin (H&E), showing villus height (VH) and crypt depth (CD), under different treatments: (**A**) treatment 1 (control); (**B**) treatment 2 (enrofloxacin 10 mg/kg); (**C**) treatment 3 (TSP 1 × MIC); (**D**) treatment 4 (TSP 16 × MIC); and (**E**) treatment 5 (TSP 32 × MIC). Morphometric data presented in Table 7. Representative sections from TSP-supplemented groups show difference in VH and CD compared with the control group, consistent with the quantitative morphometric analysis. Magnification: ×40.

**Figure 2:**
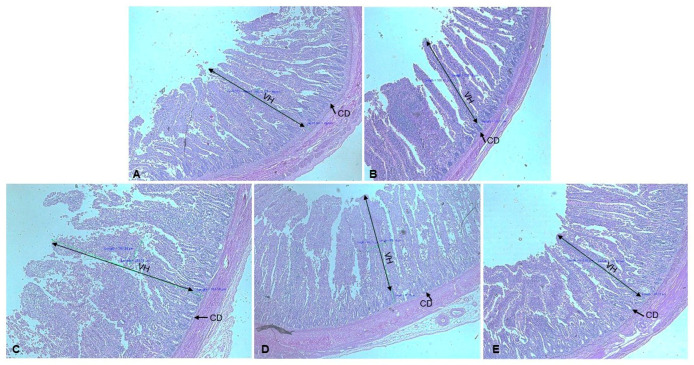
Histopathological sections of jejunum in broiler chickens stained with hematoxylin and eosin (H&E), showing villus height (VH) and crypt depth (CD), under different treatments: (**A**) treatment 1 (control); (**B**) treatment 2 (enrofloxacin 10 mg/kg); (**C**) treatment 3 (TSP 1 × MIC); (**D**) treatment 4 (TSP 16 × MIC); and (**E**) treatment 5 (TSP 32 × MIC). Morphometric data presented in Table 7. Representative sections from TSP-supplemented groups showed difference in VH and CD compared with the control group, consistent with the quantitative morphometric analysis. Magnification: ×40.

**Figure 3:**
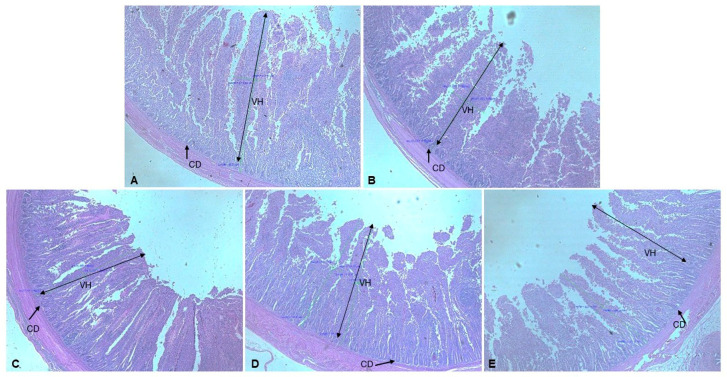
Histopathological sections of ileum in broiler chickens stained with hematoxylin and eosin (H&E), showing villus height (VH) and crypt depth (CD), under different treatments: (**A**) treatment 1 (control); (**B**) treatment 2 (enrofloxacin 10 mg/kg); (**C**) treatment 3 (TSP 1 × MIC); (**D**) treatment 4 (TSP 16 × MIC); and (**E**) treatment 5 (TSP 32 × MIC). Morphometric data presented in Table 7. Representative sections from TSP-supplemented groups show no difference in CD compared with the control group, consistent with the quantitative morphometric analysis. Magnification: ×40.

**Figure 4:**
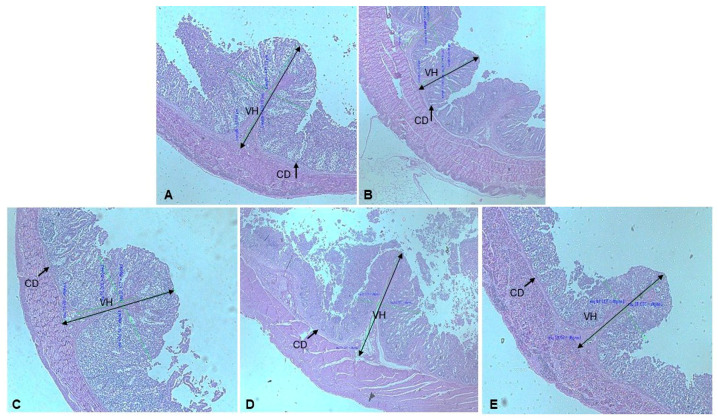
Histopathological sections of cecum in broiler chickens stained with hematoxylin and eosin (H&E), showing villus height (VH) and crypt depth (CD), under different treatments: (**A**) treatment 1 (control); (**B**) treatment 2 (enrofloxacin 10 mg/kg); (**C**) treatment 3 (TSP 1 × MIC); (**D**) treatment 4 (TSP 16 × MIC); and (**E**) treatment 5 (TSP 32 × MIC). Morphometric data presented in Table 7. Representative sections from 1 × MIC and 32 × MIC TSP-supplemented groups show difference in CD compared with the control group, consistent with the quantitative morphometric analysis. Magnification: ×40.

Morphometric assessments of duodenal villus height and jejunal and ileal villus width in broiler chickens demonstrated significant differences between the control and experimental groups at the tissue structural level on day 42 of the feeding trial, as detailed in Table 7. Specifically, the duodenal villi in all TSP groups were significantly reduced in all TSP-supplemented groups compared with the control group, although their basal widths did not differ significantly (*p* > 0.05). Furthermore, ileal villi in treatment 5 exhibited significantly greater width than those in other experimental groups (*p* < 0.05), while jejunal villi reached their maximum width in treatment 4. Regarding crypt morphology, ileal crypt depth was significantly increased compared with the control group. The duodenal crypt depth was significantly reduced in treatment 5 (TSP 32 × MIC), whereas the jejunal crypt depth did not differ from the control group (*p* < 0.05). Representative histological images illustrating the intestinal morphology of broilers from all experimental groups are presented in Figure 1, Figure 2, Figure 3 and Figure 4.

This study further demonstrated that, on day 42 of the experimental period, there was a statistically significant difference in the villus-height-to-crypt-depth ratio between the control and experimental groups across the duodenum, jejunum, and ileum (*p* < 0.05). Additionally, the ileal absorptive surface area was significantly increased in treatments 2 (enrofloxacin) and 5 (TSP 32 × MIC), whereas it was significantly decreased in treatment 4 (TSP 16 × MIC) compared with the control group (*p* < 0.05).

### 3.6. Investigation of Pathogenic Bacteria and Lactic Acid Bacteria

This study focused on the isolation and characterization of pathogenic and lactic acid bacteria from the gastrointestinal tract of broilers, employing standardized analytical methodologies (Table 8). Pathogenic bacteria were initially cultured on TSA, followed by subsequent classification using XLD agar and EMB agar. Presumptive Salmonella species were identified based on characteristic colony morphology—red colonies with black centers on XLD agar caused by the reduction of sodium thiosulfate to hydrogen sulfide (H_2_S). Meanwhile, presumptive *E. coli* was distinguished by metallic green sheen colonies on EMB agar. Colony counts were performed for each identified presumptive pathogen. Additionally, representative colonies were preserved for subsequent biochemical assays and molecular analyses to confirm their identities, as detailed in Table 9. The experimental findings indicated that groups receiving TSP 16 × MIC and TSP 32 × MIC of tamarind shell powder supplementation exhibited a lower relative proportion of pathogenic bacteria compared with the control group. Conversely, treatment 3 (TSP 1 × MIC) demonstrated a higher percentage of pathogenic bacteria relative to treatments 4 and 5. 

Probiotic bacteria were enumerated using MRS agar, a selective culture medium formulated obviously to facilitate the proliferation of Lactobacillus and other lactic acid bacteria. The findings indicated that the proportion of lactic acid bacteria was highest in the treatment with TSP 16 × MIC, particularly during period 2, when compared to the control group.

### 3.7. Pathogenic Bacteria Identification

Based on the isolation of pathogenic microorganisms utilizing XLD and EMB selective media, followed by classification through biochemical assays, the findings indicated a total of 22 isolates presumptively identified as belonging to the family Enterobacteriaceae. Subsequently, isolates designated as S19 and E20, corresponding to isolates numbered 19 and 20, respectively, were selected as representative samples for subsequent molecular characterization.

### 3.8. Probiotic Bacteria Identification

A total of 305 bacterial isolates were obtained from the duodenum, jejunum, ileum, cecum, and colon of broiler gastrointestinal tracts, as detailed in Table 10. These isolates were categorized into two chronological periods: day 21, comprising 64.00% (*n* = 144) of the samples, and day 42, comprising 71.56% (*n* = 161). Day 21, treatment 4 exhibited the highest percentages of LAB across all intestinal sections, whereas treatment 5 showed the lowest LAB prevalence. In day 42, no significant differences in LAB percentages were observed among the experimental groups.

All LAB isolates were preliminarily classified according to colony morphological features, encompassing parameters such as size, shape, coloration, and margin characteristics. Then, the isolates were grouped into 16 distinct categories, with one representative isolate from each group selected for further analysis. These selected isolates underwent a series of functional assays, including catalase activity testing, assessments of acid tolerance at pH 2.5 and pH 3.0, bile salt resistance, and gastric juice and intestinal fluid tolerance, as well as evaluations of their capacity to digest proteins, starches, and fats (Table 11).

Among 16 LAB isolates analyzed, eight isolates (M1, M5, M6, M7, M12, M13, M14, and M16) maintained viability after a four-hour incubation at pH 3, except for isolate M6, which was non-viable under these conditions. These eight isolates were subsequently subjected to evaluation in simulated gastrointestinal tract (GIT) conditions. The results revealed that seven of these isolates could withstand the simulated GIT environment, with isolate M1 being exception. Concerning nutrient hydrolysis, isolates M5 and M7 did not achieve complete degradation of all tested substrates. In contrast, isolates M1, M13, and M14 demonstrated capabilities to hydrolyze both starch and fat substrates. Additionally, isolates M6 and M12 exhibited digestibility of starch, fat, and proteins. Isolate M16 was limited to starch digestion only. The catalase activity assay indicated that isolates M1, M5, M7, M12, and M16 tested negative, whereas isolates M6, M13, and M14 tested positive for catalase activity.

### 3.9. Anti-Pathogenic Bacteria Activity of Lactic Acid Bacteria Isolated from Intestine of Broilers

Eight isolated LAB were examined for antibacterial activity using the cross-streak method. The isolated M1, M5, and M12 demonstrated against all pathogens, including *S. typhimurium*, *S. enteritidis*, *E coli*, pathogen 20 (E20), and pathogen 19 (S19). The isolated M6 and M13 showed antibacterial efficacy against pathogen 19 (S19), *S. typhimurium*, *S. enteritidis*, and *E. coli*. Pathogen 20 (E20), *S. enteritidis*, *E. coli*, and pathogen 19 (S19) were all suppressed by the isolated M7. *S. enteritidis*, *E. coli*, and pathogen 19 (S19) were all suppressed by the isolated M14. However, the isolated M16 did not exhibit inhibitory efficacy against any pathogenic strain (Table 12). Finally, isolated M1 and M12 were considered for identification by the molecular technique.

### 3.10. Molecular Identification of Pathogenic and Probiotic Bacteria

**Figure 5:**
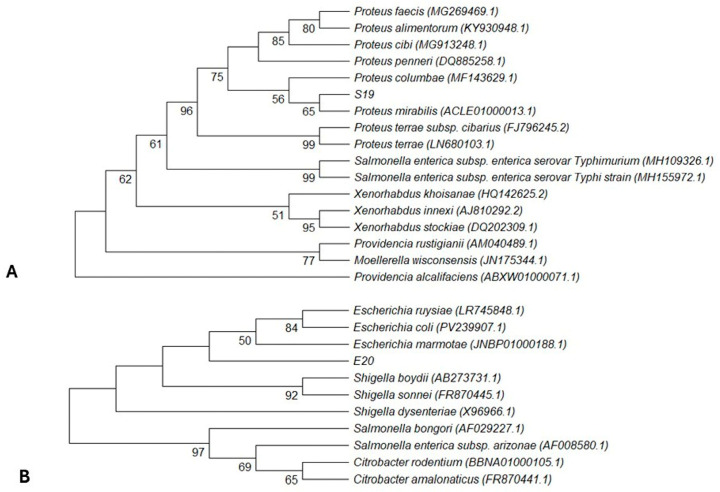
Unrooted phylogenetic tree of pathogenic bacteria in the broilers’ intestines, constructed using a neighbor-joining method. Bootstrap values for 1000 trees are shown at branch points. Only values of 50% or above are shown; (**A**) S19; (**B**) E20.

Two pathogenic bacterial strains and two probiotic bacterial strains were selected for molecular characterization and phylogenetic analysis. The complete 16S rRNA gene sequences (~1500 bp) of these strains were obtained. Nucleotide BLAST (Nucleotide BLAST at NCBI) searches indicated that the two pathogenic strains, designated S19 and E20, belong to the family Enterobacteriaceae. Strain S19 exhibited 100% sequence similarity to *Proteus mirabilis*, while strain E20 demonstrated 99.18% similarity to *Escherichia marmotae*. Phylogenetic trees were constructed using reference sequences retrieved from the NCBI database, and these were employed to determine phylogenetic relationships and distances (Figure 5). The findings confirmed that strains S19 and E20 were classified within the genera *Proteus* and *Escherichia*, respectively.

**Figure 6:**
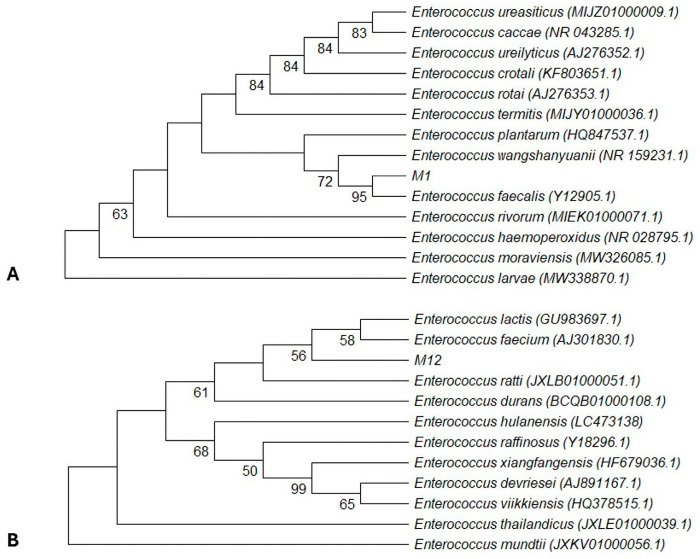
Unrooted phylogenetic tree of probiotic bacteria in the broilers’ intestines, constructed using a neighbor-joining method. Bootstrap values for 1000 trees are shown at branch points. Only values of 50% or above are shown; (**A**) M1; (**B**) M12.

The phylogenetic analysis depicted in Figure 6 demonstrates a strong genetic affiliation among the selected probiotic bacterial strains within the genus *Enterococcus*. The phylogenetic tree was generated utilizing the neighbor-joining method applied to a matrix of pairwise 16S rRNA sequence similarities. The findings indicated that isolate M1 was closely related to *Enterococcus faecalis* strains, whereas isolate M12 exhibited a close genetic relationship with *Enterococcus faecium*.

## 4. Discussion

In recent years, the feed industry has increasingly acknowledged the potential benefits of applications of plant-derived compounds across various animal species. Phytogenic feed additives (PFAs), consisting of essential oils, spices, herbs, and botanical extracts, include bioactive compounds and flavoring constituents [8], offering a multifaceted approach to enhancing animal nutrition and health [42]. Tamarind is known for its considerable nutritional and medicinal value. The fruit of tamarind consists approximately of 55% pulp, 34% seeds, and 11% shell components [43]. Tamarind has been documented to exhibit a range of pharmacological activities, including laxative and anti-diabetic effects, anti-inflammatory properties, cholesterol reduction, anti-obesity effects, antifungal activity, antioxidant capacity, antipyretic effects, and antimicrobial properties. Furthermore, it possesses appetizing and stimulatory effects that facilitate the digestive process [44]. Typically, alternative components of the tamarind tree, such as leaves or pulp, are utilized as supplemental ingredients in broiler feed formulations. This research focused on tamarind shells as a supplement in broiler chicken feed due to their status as an agricultural by-product product. However, tamarind shell retains a substantial composition of vital constituents, including insoluble dietary fiber (IDF), soluble dietary fiber (SDF), and antioxidant and antimicrobial polyphenols [45]. Moreover, there is a limited amount of research concerning the inclusion of tamarind shell as a supplement in broiler chicken diets.

Previous research has demonstrated that extracts derived from various parts of the tamarind possessed significant antibacterial properties against pathogenic bacterial strains [46]. Accordingly, the present study evaluated the antibacterial efficacy of tamarind shell extract against bacterial pathogens affecting poultry, such as *Salmonella typhimurium*, *S. enteritidis*, and *E. coli*. The results have been designed to provide guidelines for the ideal tamarind shell inclusion rate in broiler chicken feed. This research has demonstrated that tamarind shell extract exhibited in vitro antibacterial properties effective against *S. typhimurium*, *S. enteritidis*, and *E. coli* when applied at a concentration of 4 mg/mL, suggesting potential antimicrobial activity under laboratory conditions. Despite tamarind shell extract exhibiting a higher MIC compared to the antibiotic enrofloxacin (0.0005–0.0024 mg/mL), the utilization of natural active compounds in poultry feed offers several advantages, including economic benefits; reduced adverse effects on the health and growth performance of the chickens; and a decreased use of antibiotics, which may prevent the emergence of antibiotic resistance [12]. Based on the MIC values derived from antibacterial activity assessments; we quantified the levels of tamarind shell inclusion in poultry feed across three experimental groups. Each group received feed supplemented with tamarind shell at concentrations corresponding to 1 × MIC, 16 × MIC, and 32 × MIC. These experimental groups were evaluated in comparison to two control groups, one without any supplementation and another supplemented with enrofloxacin. From this result, it can be inferred that crude extract contains complex mixtures of active and inactive constituents, whereas enrofloxacin is a purified compound that can be used to inhibit bacterial growth. Therefore, the antibacterial activity of *Tamarindus indica* shell extract should not be considered comparable to enrofloxacin [47,48,49]. Instead, its potential value will be applied as a phytogenic feed additive that may support gut health and reduce reliance on antibiotics in poultry production. Based on the MIC values, dietary supplementation of 1 × MIC, 16 × MIC, and 32 × MIC was selected for the feeding trial to evaluate its effect on growth performance, health status, and intestinal microbial balance in broiler chickens.

Plant extracts are frequently added to poultry diets to enhance growth performance and overall animal health. Extensive research has documented the beneficial effects of plant extracts on poultry performance, indicating that supplementation with plant extracts can lead to increased BWG, ADFI, and AFCR [50]. In this study, TSP at 16 × MIC demonstrated a significant improvement of growth performance and nutrient utilization, particularly between days 1 and 21. TSP supplementation was associated with a significant enhancement in ADFI, an increase in BWG, and an improvement in ADG in poultry, relative to control groups. During day 22–42, the continued administration of TSP maintained its positive influence on ADFI and AFCR. The observed enhancements in growth performance and nutrient utilization might be attributable to the presence of beneficial phytochemicals in TSP, especially polyphenols.

Previous research has identified that *T. indica* shell extract contains phenolic compounds at a concentration of 68.67 ± 0.26 mg/g GAE, flavonoids at 57.38 ± 0.20 mg/g CE, and tannins as 86.66% of the extract [51]. These phytochemicals contribute to the development of a balanced gut microbiota and improve nutrient digestibility by stimulating the secretion of digestive enzymes. Although polyphenolic compounds are prevalent in medicinal herbs [8] and exhibit limited bioavailability in animals, they are often metabolized by gut microbiota into bioactive, beneficial compounds that possess antimicrobial, anti-inflammatory, digestive, and antioxidant properties [8,52]. *T. indica* shell powder affected growth performance in a dose- and age-dependent manner. The administration of medium (TSP 16 × MIC) improved early growth parameters, including ADFI and BWG; however, this response did not lead to better AFCR, indicating an imbalance between nutrient intake and efficiency. Similarly, results have been reported for plant-derived additives, where improved growth is not always associated with improved feed efficiency [41,42]. In the second period, treatment responses became more variable, suggesting that physiological adaptation and age-related changes in digestive and metabolic capacity may modulate the efficacy of plant-derived additives. Overall, the results indicate that TSP supplementation exerts different effects depending on dose and growth stage [41,42,43].

Hematological parameters serve as crucial indicators for assessing feed toxicity, because dietary constituents may influence blood physiology and overall health status in farm animals. The present study demonstrated notable elevations in PCV, RBC count, and WBC count. Consistent with these findings, prior research has reported similar hematological enhancements in broilers supplemented with *Lepidium sativum* seed powder [34], *Capsicum frutescens*, and *Curcuma longa* powders [40], as well as pawpaw (*Asimina triloba*) leaf and seed meal [53], indicating a pattern of increased PCV and RBC values associated with these dietary interventions. The health status of poultry can be assessed through the measurement of PCV. A PCV value below 35% is indicative of anemia, whereas a value exceeding 55% may suggest the presence of hyperglycemia or dehydration. Furthermore, PCV plays a crucial role in the facilitation of oxygen transport and nutrient absorption [54,55]. In the present study, the PCV values observed in treatment 5 (TSP 32 × MIC) consistently surpassed 55% over a 21-day period, which may be considered excessively high for broilers during their early developmental stages. On day 21, treatments 4 (TSP 16 × MIC) and 5 (TSP 32 × MIC) had greater WBC counts, and on day 42, they were almost identical to the control group. High WBC counts in animals may reflect an illness and can lead to the production of antibodies during phagocytosis [56,57]. Overall, this study observed higher values of hematological parameters, especially PCV and WBC counts in TSP-supplemented groups. These changes may be related to physiological responses to bioactive compounds in the broiler diet, as no clinical signs of toxicity were detected during the trial period [37,40,53]. However, the elevated PCV in treatment 5 (TSP 32 × MIC) should be carefully interpreted, as values exceeding the normal physiological range may indicate alterations in blood volume status [54]. The modulation of leukocyte profiles, including increased WBC and lymphocyte proportions, suggests a potential influence of TSP supplementation on immune-related responses in broiler chickens [56,58].

Serum biochemical parameters of broiler chickens administered with TSP exhibited variations. The findings indicated that ALT concentrations were significantly affected (*p* < 0.05) by different concentrations of TSP in comparison to the control group. ALT is a hepatic-specific enzyme that serves as a biomarker for liver injury. It is predominantly localized within hepatocytes; however, upon hepatic injury or damage, ALT is released into the bloodstream, signaling hepatic dysfunction. Elevated serum ALT levels are indicative of ongoing hepatocellular damage [56]. In the present study, no significant differences in ALT levels were observed on day 21. However, on day 42, ALT concentrations decreased through all treatment groups compared to the control, suggesting a modulation of hepatocellular enzyme leakage under physiological conditions rather than evidence of liver injury or recovery, as all values remained within the normal physiological range of broiler chickens [40]. Additionally, these results demonstrated that TSP supplementation decreased total cholesterol but had no significant effect on triglycerides. The decrease in serum cholesterol observed in laying hens administered tamarind may also be attributed to a decline in the activity of biosynthetic enzymes [57]. These results align with prior studies. Chowdhury et al. [57] observed that the feeding of 2, 4, 6, or 8% oven-dried and crushed tamarind decreased serum cholesterol concentrations by 12 to 14%. Overall, these findings indicate that TSP supplementation may modulate serum biochemical parameters related to hepatic function and lipid metabolism without inducing adverse physiological effects under the condition of this study.

The health of the poultry gastrointestinal tract has received considerable research interest in recent years, primarily due to the poultry industry’s shift away from incorporating therapeutic antibiotics into feed regimens [59]. Elevated intestinal permeability resulting from various stressors can adversely affect poultry performance and productivity while also compromising overall health status [60]. The assessment of intestinal permeability is typically conducted using 4 kDa of fluorescein isothiocyanate-conjugated dextran (FITC–dextran), a non-digestible dextran conjugated with a fluorescent label that has been validated as an appropriate marker for evaluating intestinal barrier function in avian species [61]. FITC–dextran can pass through damaged epithelial barriers after oral delivery, allowing its bloodstream concentration to be determined. As a biomarker for cell permeability, serum FITC–dextran levels indicate the degree and severity of intestinal mucosal barrier failure and reflect the cumulative translocation of the marker into systemic circulation, whereas tissue-related FITC–dextran represents local retention within specific intestinal segments at the time of sampling [60]. The observed differences of intestinal segments (duodenum, jejunum, ileum, and cecum) likely reflect absorptive function, epithelial structure, and microbial density in the gastrointestinal tract [25,27,59]. This investigation demonstrated that supplementation of broiler chicken diets with TSP increased serum FITC-d levels in treatment 5 (TSP 32 × MIC) when compared to the control group. Meanwhile, treatments 3 (TSP 1 × MIC) and 4 (TSP 16 × MIC) showed no significant differences in FITC–dextran levels in both serum and intestine. The pattern observed, particularly the elevation in serum FITC–dextran in the absence of consistent intestinal accumulation, may suggest a time-dependent translocation of FITC–dextran across the intestinal epithelium. However, this study was not conducted with a disease-challenge model, so these findings should be interpreted as modulation of intestinal permeability rather than direct evidence of the intestinal inflammation or pathological barrier disruption [25,27,59]. Overall, these results indicate that TSP supplementation may influence gut barrier function in a dose-dependent manner, particularly at higher inclusion levels. However, further study under disease-challenge conditions is required to clarify its functional role in intestinal health.

Maintaining intestinal morphology is essential for nutrient digestion and absorption in animals. The gastrointestinal system plays an important role in feed intake and the efficient absorption of nutrients in farm animals. Villus height, crypt depth, and the villus-to-crypt ratio of the duodenum, jejunum, and ileum are commonly used indicators for evaluating intestinal morphology in broiler chickens, particularly in this study investigating dietary interventions and feed additives [62]. Villus height is commonly used for evaluating the absorptive surface area of the intestine and brush border enzyme activity [63]. In the present study, dietary supplementation with TSP supplementation resulted in the segment-dependent changes in intestinal morphology. A reduction in villus height was observed in the duodenum of all TSP-treated groups compared with the control group, whereas villus width and crypt depth showed variable responses depending on the intestinal segment and TSP-supplemented level. In contrast, the jejunum and ileum exhibited morphological changes, including variation in villus width, crypt depth, and villus-to-crypt ratio depending on TSP-supplemented level [64,65]. The villus-to-crypt ratio, an indicator of the villus-to-crypt ratio, an indicator of the balance between absorptive surface area and epithelial turnover, was altered in a dose- and segment-dependent manner. This study was notably elevated in the duodenum of broilers administered 32 × MIC of TSP. Additionally, this ratio was enhanced in the jejunum at 16 × MIC of TSP and in the cecum in all tested TSP supplementations; these findings may indicate alteration of absorptive efficiency, while reductions in other segments indicate localized adaptive responses of the intestinal epithelium. TSP supplementation may modulate intestinal morphology in a region-specific manner rather than affecting the entire gastrointestinal tract. Such variability may be attributed to differences in physiological function, epithelial turnover, microbial density, and nutrient absorption capacity along the small intestine [66,67]. Previous studies have reported that tamarind-derived products can influence growth performance and gut-related parameters in broiler chickens [68,69,70]. Nevertheless, the present study was not conducted under a disease-challenge model; the observed morphological changes should be interpreted as adaptive responses of intestinal tissue rather than direct evidence of improved intestinal health status. Broiler heat stress may be reduced by the polyphenolic chemicals found in *T. indica* leaf extracts. Proteins; fibers; lipids; and several vitamins, such as B1, B2, B3, vitamin C, and β-carotene, as well as flavonoids and polyphenols, which have antibacterial qualities, are abundant in *T. indica* leaves. Moreover, β-carotene is a precursor to the synthesis of vitamin A, while vitamin C is an antioxidant [71,72].

The assessment of gut health following TSP supplementation revealed that TSP effectively promoted the increase in LAB and suppressed pathogenic bacterial species throughout both experimental phases, notably in treatments 4 (TSP 16 × MIC) and 5 (TSP 32 × MIC). An increase in LAB was associated with enhanced broiler health, because LAB was linked to facilitating digestive processes, optimizing nutrient absorption, and strengthening the avian immune response. Furthermore, probiotics can inhibit the proliferation of pathogenic microorganisms within the gastrointestinal tract through competitive exclusion for adhesion sites and nutrients [73]. In this investigation, two LAB strains were chosen as representatives and identified using molecular methods. Strain M1 was closely associated with *E. faecalis*, while strain M12 was closely related to *E. faecium*. Enterococci are probiotic bacteria that naturally inhabit the gastrointestinal tracts of animals. LAB produced bioactive metabolites with significant health benefits for broilers. These bioactive metabolites included organic acids (lactic and acetic acid), bacteriocins, hydrogen peroxide, and exopolysaccharides, which exert antimicrobial properties against pathogenic bacteria, promoting a balanced intestinal microbiome and improving overall health in broilers [74]. The bacteriocins produced by enterococci encompass well-characterized compounds, such as enterocins, mundticin, bacteriocin, and enterolysin [75]. Bacteriocins are able to have a bacteriostatic impact by preventing the synthesis of proteins, DNA, and RNA, or they can penetrate cell membranes and leak cell contents like K^+^ and inorganic phosphate [76].

Gut microbiota is essential for intestine defense, host immunity, and nutritional absorption. On the other hand, intestinal pathogen infections cause gut inflammation and poor developmental performance. In this study, the molecular analysis identified *P. mirabilis* and *E. coli* as the predominant pathogenic bacterial strains in the control group that showed diarrhea symptoms. In avian species, *E. coli* infection is common [77]. Avian colibacillosis can manifest as airsacculitis, pericarditis, pneumonia, and hepatitis, which significantly impair production [78]. Evidence has emerged suggesting that pathogenic *E. coli* co-infections with other gut bacteria, such as *Proteus* mirabilis, may exacerbate disease severity through mechanisms that are not yet fully understood [78,79,80]. In avians with colibacillosis, alterations in the gut microbiota have been detected when compared to healthy flocks [80]. Imbalanced microbiota not only results in higher opportunistic pathogens but also provides antibiotic resistance [78]. Importantly, with these conditions, the relative abundance of *Proteus* spp. significantly increases [78]. *Proteus mirabilis* is a Gram-negative bacillus that is commonly found in various environments, including in the gastrointestinal tract of both humans and animals [81,82]. It is a significant opportunistic pathogen responsible for a wide range of infections and contributes to inflammation in the gastrointestinal tract [78,82]. Carvajal et al. [83] reported that *E. faecium* and *E. faecalis* exhibited probiotic activity, positively influencing growth performance and pathogen inhibition. The co-infection with these pathogenic bacteria can induce diarrhea and disrupt the gut barrier by triggering inflammation of the gastrointestinal tract in broiler chickens [78]. In this investigation, neither the antibiotic treatment group nor the chickens administered medium or high dosages of TSP had any *E. coli* infections. During the early phases of chicken growth, some H_2_S-producing bacteria were still developing, but their numbers were lower than those in the control group. Overall, TSP supplementation maintained the intestinal microbial balance and improved gut health by promoting the growth of beneficial bacteria like LAB and inhibiting *E. coli* and *Salmonella* infection in broilers’ gastrointestinal tracts.

## 5. Conclusions

The poultry industry is actively seeking safe and effective growth promoters, with tamarind shell emerging as a potential candidate. Tamarind shell possesses a variety of valuable properties that render it suitable for multiple applications. The findings of this study demonstrated a positive correlation between broiler chicken growth performance, health status, and dietary levels of tamarind shell powder (TSP). Specifically, TSP administered at 16 × MIC as a natural dietary supplement may serve as a beneficial strategy to enhance health parameters and growth indicators, including weight gain and feed intake. Additionally, TSP contributed to the balancing of the gastrointestinal microbiota by promoting the proliferation of probiotic bacteria, while concurrently suppressing the proliferation of pathogenic microorganisms. These results suggest that tamarind shells represent a potential natural feed additive to support growth performance and intestinal health, and reduce reliance on synthetic additives and antibiotics in broiler chickens.

## Figures and Tables

**Table 1:** Ingredients and nutrient composition of the experimental diets for broiler chickens (1–42 days).

Ingredients	Phase			
	1–10 Days	11–24 Days	25–39 Days	40–42 Days
Corn meal (kg)	52.83	58.00	59.50	60.00
Soybean meal (kg)	32.00	27.33	27.33	26.83
Fish meal (kg)	8.00	6.00	5.50	5.00
Palm oil (kg)	5.00	6.50	5.00	6.00
Salt (kg)	0.30	0.3	0.30	0.30
Dicalcium phosphate (kg)	1.00	1.00	1.00	1.00
Combo mix ^a^ (kg)	0.50	0.50	1.00	0.50
DL-methionine (kg)	0.12	0.12	0.12	0.12
L-lysine (kg)	0.25	0.25	0.25	0.25
**Total (kg)**	100	100	100	100
**Nutritional compositions (% dry matter)**				
Crude protein (%)	23	21.5	19.5	18
Metabolizable energy (Kcal/kg)	3000	3100	3200	3225
Lysine (%)	1.44	1.29	1.15	1.08
Methionine (%)	0.56	0.51	0.47	0.44
Methionine + cystine (%)	1.08	0.99	0.90	0.85
Threonine (%)	0.97	0.88	0.78	0.73
Calcium (%)	0.96	0.87	0.78	0.74
Available phosphorus (%)	0.48	0.44	0.39	0.37
Sodium (%)	0.16–0.23	0.16–0.23	0.16–0.23	0.16–0.23

^a^ Vitamin–mineral premix contains in the following per kg: vitamin A, 2,500,000 IU; vitamin D_3_, 500,000 IU; vitamin E, 3000 IU; vitamin K_3_, 0.20 g; vitamin B_2_, 0.08 g; vitamin B_6_, 1.40 g; vitamin B_12_, 0.002 g; niacin, 4.80 g; pantothenic acid, 4.00 g; choline, 200.00 g; folic acid, 0.12 g; zinc, 16.00 g; iron, 30.80 g; manganese, 24.00 g; copper, 2.20 g; cobalt, 0.06 g; iodine, 0.14 g.

**Table 2:** Antibacterial activity of tamarind shell extract.

Compound	MIC/MBC Concentration (mg/mL)		
	*S. typhimurium*	*S. enteritidis*	*E. coli*
Tamarind shell extract	4/64	4/64	4/8
Enrofloxacin	0.0024/0.0024	0.0005/0.0313	0.0012/0.0012

**Table 3:** Effects of different dietary tamarind shell powder supplementations on the growth performance and feed efficiency parameters of broiler chickens. TSP, tamarind shell powder; ADFI, average daily feed intake; BWG, body weight gain; ADG, average daily gain; AFCR, average feed conversion ratio.

Parameter	Period	Productive Index (Mean ± S.E.)				
		Treatment 1 (Control)	Treatment 2 (Enrofloxacin)	Treatment 3 (TSP 1 × MIC)	Treatment 4 (TSP 16 × MIC)	Treatment 5 (TSP 32 × MIC)
ADFI (g/day/chick)	1–21 days	72.51 ± 2.15 ^b^	65.57 ± 3.03 ^a^	73.54 ± 0.49 ^b^	85.38 ± 2.03 ^c^	76.56 ± 1.26 ^b^
	22–42 days	170.29 ± 2.02 ^a^	226.54 ± 11.45 ^bc^	184.38 ± 12.78 ^a^	257.52 ± 4.03 ^c^	198.95 ± 16.52 ^ab^
	1–42 days	102.29 ± 9.71 ^a^	146.05 ± 7.23 ^b^	128.96 ± 6.50 ^b^	171.45 ± 3.01 ^c^	137.76 ± 8.75 ^b^
BWG (g)	1–21 days	651.38 ± 22.98 ^ab^	723.20 ± 22.87 ^abc^	698.94 ± 11.60 ^bc^	787.27 ± 24.23 ^c^	763.01 ± 36.36 ^a^
	22–42 days	1370.26 ± 54.27 ^a^	1440.37 ± 30.87 ^a^	1869.96 ± 171.56 ^b^	1580.33 ± 84.37 ^a^	1409.67 ± 22.85 ^a^
	1–42 days	2021.65 ± 36.08	2163.57 ± 46.47	2149.12 ± 74.26	2226.16 ± 53.31	2172.68 ± 58.69
ADG (g/day/chick)	1–21 days	31.02 ± 1.10 ^a^	34.44 ± 1.09 ^abc^	33.28 ± 0.55 ^ab^	37.49 ± 1.15 ^c^	36.33 ± 1.73 ^bc^
	22–42 days	65.25 ± 2.58	68.59 ± 1.47	69.06 ± 3.01	68.52 ± 3.20	67.13 ± 1.09
	1–42 days	48.13 ± 0.86	51.51 ± 1.11	51.17 ± 1.77	53.00 ± 1.27	51.73 ± 1.40
AFCR	1–21 days	2.34 ± 0.08 ^b^	1.91 ± 0.14 ^a^	2.21 ± 0.03 ^b^	2.28 ± 0.03 ^b^	2.12 ± 0.10 ^a^
	22–42 days	2.62 ± 0.12 ^ab^	3.31 ± 0.23 ^c^	2.05 ± 0.06 ^a^	3.45 ± 0.22 ^c^	2.96 ± 0.21 ^bc^
	1–42 days	2.12 ± 0.19 ^a^	2.84 ± 0.20 ^bc^	2.53 ± 0.18 ^ab^	3.24 ± 0.12 ^c^	2.66 ± 0.13 ^ab^
Mortality rate (%)	42 days	4.67 ± 0.33 ^c^	2.00 ± 0.00 ^a^	3.33 ± 0.33 ^b^	2.33 ± 0.33 ^a^	1.67 ± 0.33 ^a^

^a,b,c^: mean ± S.E. with different superscripts in the same row are significantly different at *p* < 0.05.

**Table 4:** Hematological parameters in broilers administered different levels of tamarind shell powder. An analysis at days 21 and 42. TSP, tamarind shell powder; PCV, packed cell volume; RBC, red blood cell count; WBC, white blood cell count.

Blood Parameters	Period	Treatment 1 (Control)	Treatment 2 (Enrofloxacin)	Treatment 3 (TSP 1 × MIC)	Treatment 4 (TSP 16 × MIC)	Treatment 5 (TSP 32 × MIC)
PCV (%)	21 days	47.67 ± 0.94 ^a^	56.87 ± 2.08 ^b^	46.24 ± 0.77 ^a^	49.14 ± 1.62 ^a^	63.41 ± 1.94 ^c^
	42 days	46.05 ± 0.54 ^b^	38.99 ± 0.36 ^a^	45.10 ± 0.28 ^b^	53.81 ± 0.71 ^c^	53.10 ± 0.52 ^c^
RBC count (×10^6^ cells/mm^3^)	21 days	1.72 ± 0.13 ^b^	1.29 ± 0.08 ^a^	2.50 ± 0.14 ^c^	1.34 ± 0.08 ^a^	1.57 ± 0.13 ^ab^
	42 days	1.28 ± 0.09	1.10 ± 0.10	1.23 ± 0.06	1.10 ± 0.03	1.09 ± 0.06
WBC count (×10^6^ cells/mm^3^)	21 days	1.34 ± 0.00 ^b^	1.37 ± 0.00 ^b^	1.22 ± 0.02 ^a^	1.77 ± 0.02 ^c^	1.92 ± 0.01 ^d^
	42 days	1.41 ± 0.01 ^d^	0.90 ± 0.00 ^c^	0.97 ± 0.00 ^e^	1.16 ± 0.00 ^a^	1.35 ± 0.00 ^b^
Differential WBC (%)						
Lymphocytes	21 days	35.15 ± 0.83 ^a^	45.28 ± 1.83 ^b^	46.61 ± 1.99 ^b^	45.78 ± 1.43 ^b^	43.74 ± 1.20 ^b^
	42 days	45.76 ± 1.59	49.07 ± 2.18	46.61 ± 1.24	50.67 ± 2.17	49.42 ± 1.39
Monocytes	21 days	6.33 ± 0.79 ^b^	4.98 ± 0.45 ^ab^	4.76 ± 0.57 ^ab^	3.74 ± 0.63 ^a^	4.52 ± 0.33 ^a^
	42 days	5.19 ± 0.50 ^ab^	5.61 ± 0.50	3.93 ± 0.39 ^a^	5.02 ± 0.59 ^ab^	6.20 ± 0.49 ^b^
Eosinophils	21 days	5.78 ± 0.52 ^ab^	7.94 ± 0.77 ^c^	5.56 ± 0.39 ^ab^	4.78 ± 0.63 ^a^	7.61 ± 1.02 ^bc^
	42 days	6.19 ± 0.46 ^b^	5.69 ± 0.49 ^b^	3.80 ± 0.34 ^a^	4.07 ± 0.73 ^a^	5.76 ± 0.71 ^b^
Heterophils	21 days	50.44 ± 0.62 ^c^	39.30 ± 1.81 ^a^	41.04 ± 1.59 ^ab^	44.26 ± 1.48 ^b^	41.74 ± 1.72 ^ab^
	42 days	41.04 ± 1.67	38.69 ± 1.60	42.26 ± 1.21	37.79 ± 1.48	37.20 ± 1.25
Basophils	21 days	2.39 ± 0.34 ^b^	2.30 ± 0.26 ^b^	1.94 ± 0.20 ^ab^	1.41 ± 0.23 ^a^	2.13 ± 0.18 ^ab^
	42 days	1.83 ± 0.28 ^bc^	0.91 ± 0.26 ^a^	3.41 ± 0.36 ^d^	2.44 ± 0.28 ^c^	1.44 ± 0.22 ^ab^

^a,b,c,d^: mean ± S.E. with different superscripts in the same row are significantly different at *p* < 0.05.

**Table 5:** Serum biochemical indices of broiler chicken fed diet supplemented with tamarind shell powder. TSP, tamarind shell powder; ALT, alanine transaminase; ALP, alkaline phosphatase; A:G ratio, the albumin-to-globulin ratio.

Serum Biochemical Parameters		Treatment 1 (Control)	Treatment 2 (Enrofloxacin)	Treatment 3 (TSP 1 × MIC)	Treatment 4 (TSP 16 × MIC)	Treatment 5 (TSP 32 × MIC)
ALT (U/L)	21 days	2.11 ± 0.20	1.56 ± 0.24	2.50 ± 0.44	2.22 ± 0.22	2.22 ± 0.22
	42 days	2.56 ± 0.34 ^bc^	2.00 ± 0.33 ^c^	0.89 ± 0.26 ^a^	0.89 ± 0.26 ^a^	1.44 ± 0.24 ^ab^
Creatinine (mg/dL)	21 days	0.39 ± 0.01 ^b^	0.34 ± 0.02 ^a^	0.39 ± 0.01 ^b^	0.40 ± 0.00 ^b^	0.37 ± 0.02 ^ab^
	42 days	0.39 ± 0.02 ^b^	0.33 ± 0.03 ^ab^	0.37 ± 0.02 ^ab^	0.29 ± 0.03 ^a^	0.29 ± 0.05 ^a^
ALP (U/L)	21 days	22,941.56 ± 2095.43	23,361.11 ± 2141.58	17,582.22 ± 2445.92	22,023.78 ± 1157.03	24,962.33 ± 2088.02
	42 days	6550.22 ± 1772.70	7990.44 ± 1972.55	9422.22 ± 3164.38	6838.00 ± 2542.97	5427.11 ± 1622.40
Cholesterol (mg/dL)	21 days	168.00 ± 3.60 ^b^	173.44 ± 3.72 ^b^	149.63 ± 4.36 ^a^	153.89 ± 4.58 ^a^	167.67 ± 4.37 ^b^
	42 days	141.00 ± 6.82	144.00 ± 8.17	119.89 ± 9.87	118.44 ± 14.12	112.11 ± 9.27
Triglyceride (mg/dL)	21 days	81.78 ± 5.80 ^a^	125.78 ± 6.68 ^b^	93.63 ± 6.58 ^a^	75.78 ± 5.78 ^a^	95.11 ± 6.41 ^a^
	42 days	26.67 ± 2.77	25.11 ± 3.38	30.44 ± 5.03	34.67 ± 4.82	25.11 ± 3.20
Albumin (g/dL)	21 days	1.28 ± 0.04 ^bc^	1.21 ± 0.03 ^ab^	1.25 ± 0.03 ^bc^	1.14 ± 0.02 ^a^	1.31 ± 0.03 ^c^
	42 days	1.22 ± 0.04	1.24 ± 0.05	1.17 ± 0.06	1.28 ± 0.10	1.11 ± 0.08
Globulin (g/dL)	21 days	1.29 ± 0.03	1.37 ± 0.03	1.36 ± 0.04	1.37 ± 0.04	1.38 ± 0.02
	42 days	1.51 ± 0.07 ^ab^	1.51 ± 0.05 ^ab^	1.33 ± 0.05 ^a^	1.66 ± 0.11 ^b^	1.33 ± 0.09 ^a^
Total protein (mg/dL)	21 days	2.57 ± 0.05	2.58 ± 0.06	2.60 ± 0.06	2.51 ± 0.05	2.58 ± 0.06
	42 days	2.73 ± 0.10	2.76 ± 0.09	2.50 ± 0.09	2.93 ± 0.20	2.76 ± 0.09
A:G ratio	21 days	0.99 ± 0.03 ^d^	0.89 ± 0.01 ^ab^	0.92 ± 0.02 ^bc^	0.84 ± 0.02 ^a^	0.89 ± 0.01 ^cd^
	42 days	0.81 ± 0.03	0.83 ± 0.03	0.88 ± 0.04	0.77 ± 0.03	0.83 ± 0.03

^a,b,c,d^: means ± S.E. with different superscripts in the same row are significantly different at *p* ≤ 0.05.

**Table 6:** Serum FITC–dextran and FITC–dextran retentions in the small intestine from different dietary supplementations with tamarind shell powder in broiler chickens.

Treatment (Mean ± S.E.)		Treatment 1 (Control)	Treatment 2 (Enrofloxacin)	Treatment 3 (TSP 1 × MIC)	Treatment 4 (TSP 16 × MIC)	Treatment 5 (TSP 32 × MIC)
FITC–dextran in serum (μg/mL)		3.30 ± 0.03 ^a^	3.37 ±0.03 ^ab^	3.38 ±0.02 ^ab^	3.42 ± 0.04 ^b^	3.57 ± 0.04 ^c^
FITC–dextran in tissues (μg/mL)	Duodenum	9.19 ± 0.52 ^bc^	8.43 ± 0.44 ^ab^	9.08 ± 0.26 ^bc^	9.63 ± 0.39 ^c^	7.65 ± 0.20 ^a^
	Jejunum	8.82 ± 0.52	7.68 ± 0.33	8.65 ± 0.50	8.78 ± 0.19	8.42 ± 0.33
	Ileum	8.54 ± 0.53 ^b^	8.55 ± 0.32 ^b^	7.26 ± 0.25 ^a^	9.00 ± 0.38 ^b^	8.05 ± 0.45 ^b^
	Cecum	13.09 ± 0.74	11.84 ± 0.50	11.93 ± 0.48	13.16 ± 0.30	11.89 ± 0.67

^a,b,c^: means ± S.E. with different superscripts in the same row are significantly different at *p* ≤ 0.05. TSP: tamarind shell powder.

**Table 7:** The values of evaluated parameters of the small intestine of broiler chickens on day 42 of the feeding trial.

Parameters (Mean ± S.E.)	Intestinal Part	Treatment 1 (Control)	Treatment 2 (Enrofloxacin)	Treatment 3 (TSP 1 × MIC)	Treatment 4 (TSP 16 × MIC)	Treatment 5 (TSP 32 × MIC)
Villus height (µm)	Duodenum	1101.13 ± 7.14 ^c^	1030 ± 137.59 ^bc^	823.92 ± 16.41 ^ab^	917.94 ± 23.81 ^abc^	773.71 ± 13.36 ^a^
	Jejunum	888.85 ± 99.09	956.91 ± 44.47	865.13 ± 9.72	951.80 ± 41.39	916.45 ± 29.32
	Ileum	959.67 ± 51.78	1082.33 ± 85.23	1075.03 ± 19.55	910.51 ± 5.67	1164.48 ± 85.24
	Cecum	635.27 ± 145.99	377.75 ± 58.44	732.46 ± 156.67	689.97 ± 157.92	768.26 ± 78.15
Villus width (µm)	Duodenum	214.13 ± 21.66	192.64 ± 49.51	150.95 ± 7.92	191.62 ± 6.33	229.54 ± 15.98
	Jejunum	196.73 ± 13.42 ^b^	131.23 ± 2.94 ^a^	152.50 ± 15.51 ^a^	237.53 ± 15.25 ^c^	158.51 ± 0.58 ^a^
	Ileum	198.39 ± 8.39 ^b^	232.49 ± 20.52 ^bc^	201.26 ± 8.86 ^b^	135.50 ± 9.71 ^a^	255.63 ± 11.64 ^c^
	Cecum	462.28 ± 24.03	476.47 ± 84.23	521.49 ± 53.53	506.27 ± 62.96	490.66 ± 13.53
Crypt depth (µm)	Duodenum	156.81 ± 3.21 ^c^	131.72 ± 9.50 ^b^	134.23 ± 0.89 ^b^	178.64 ± 3.61 ^d^	63.32 ± 0.85 ^a^
	Jejunum	98.01 ± 4.21 ^a^	107.88 ± 2.45 ^a^	124.19 ± 1.76 ^b^	104.55 ± 8.62 ^a^	131.34 ± 1.41 ^b^
	Ileum	93.94 ± 7.07 ^a^	212.30 ± 22.51 ^c^	170.73 ± 2.08 ^b^	129.60 ± 8.57 ^a^	170.54 ± 6.56 ^b^
	Cecum	119.86 ± 6.68	62.89 ± 7.95	123.62 ± 26.76	125.12 ± 45.07	136.58 ± 21.51
Villus: crypt ratio	Duodenum	7.02 ± 0.14 ^b^	7.82 ± 0.77 ^b^	6.14 ± 0.09 ^ab^	5.14 ± 0.07 ^a^	12.22 ± 0.18 ^c^
	Jejunum	9.02 ± 0.70 ^b^	8.88 ± 0.48 ^b^	6.97 ± 0.17 ^a^	9.20 ± 0.62 ^b^	6.98 ± 0.27 ^a^
	Ileum	10.25 ± 0.32 ^c^	5.18 ± 0.59 ^a^	6.30 ± 0.17 ^ab^	7.09 ± 0.46 ^b^	6.88 ± 0.75 ^b^
	Cecum	5.19 ± 0.97	6.37 ± 1.50	5.92 ± 0.22	6.09 ± 1.09	5.77 ± 0.86
Villus absorptive surface area (µm^2^)	Duodenum	0.74 ± 0.08	0.67 ± 0.26	0.39 ± 0.03	0.55 ± 0.01	0.56 ± 0.03
	Jejunum	0.55 ± 0.09 ^ab^	0.40 ± 0.01 ^a^	0.41 ± 0.04 ^a^	0.71 ± 0.07 ^b^	0.46 ± 0.01 ^a^
	Ileum	0.60 ± 0.05 ^b^	0.78 ± 0.04 ^cd^	0.68 ± 0.02 ^bc^	0.39 ± 0.03 ^a^	0.94 ± 0.09 ^d^
	Cecum	0.94 ± 0.26	0.59 ± 0.17	1.15 ± 0.16	1.16 ± 0.35	1.18 ± 0.11

^a,b,c,d^ indicate a significant difference at 0.05 in column. TSP: tamarind shell powder.

**Table 8:** Percentage of pathogenic and lactic acid-producing bacterial population within the gastrointestinal microbiota of broiler chickens subjected to various treatment groups.

Treatment (Mean ± S.E.)	Period	Treatment 1 (Control)	Treatment 2 (Enrofloxacin)	Treatment 3 (TSP 1 × MIC)	Treatment 4 (TSP 16 × MIC)	Treatment 5 (TSP 32 × MIC)
H_2_S-produced forming colonies (%)	21 days	12.83 ± 4.28	0.00 ± 0.00	14.81 ± 2.14	11.11 ± 3.70	11.11 ± 3.70
	42 days	3.70 ± 2.14	0.00 ± 0.00	7.78 ± 4.17	3.70 ± 2.14	3.70 ± 2.14
*Escherichia coli* (%)	21 days	14.81 ± 2.14	11.11 ± 3.70	3.70 ± 2.14	0.00 ± 0.00	0.00 ± 0.00
	42 days	7.41 ± 4.28	0.00 ± 0.00	0.00 ± 0.00	0.00 ± 0.00	0.00 ± 0.00
Lactic acid bacteria (%)	21 days	22.22 ± 0.00	25.93 ± 2.14	22.22 ± 0.00	25.93 ± 2.14	22.22 ± 0.00
	42 days	14.81 ± 2.14	14.81 ± 2.14	18.52 ± 5.66	33.33 ± 0.00	25.93 ± 4.28

**Table 9:** Biochemical test of pathogenic bacteria from broiler chickens.

Sample	TSI Slant/Butt/Gas	MIL Motile/LDC	Simmon Citrate Agar Slant/Butt	LIA Slant/Butt/Gas
*Salmonella*	Alkaline/acid/gas with H_2_S	+/+	+	Alkaline/alkaline/gas
*E. coli*	Acid/acid/gas	+/+	−	Alkaline/alkaline/gas
1	Acid/acid/gas	+/+	−	Alkaline/acid
2	Acid/acid/gas	+/+	−	Alkaline/acid
3	Acid/acid/gas	+/+	+	Alkaline/alkaline/gas
4	Alkaline/acid/gas with H_2_S	+/+	+	Alkaline/acid/gas
5	Acid/acid/gas	+/+	−	Acid/acid/gas
6	Alkaline/acid/gas with H_2_S	+/−	−	Alkaline/alkaline/gas
7	Alkaline/acid/gas with H_2_S	+/+	−	Alkaline/alkaline/gas
8	Alkaline/acid/gas with H_2_S	+/+	−	Alkaline/alkaline/gas
9	Alkaline/acid/gas with H_2_S	+/+	−	Acid/acid/gas
10	Alkaline/acid/gas with H_2_S	+/+	−	Alkaline/alkaline/gas
11	Acid/alkaline/gas	+/−	+	Acid/acid/gas
12	Alkaline/acid/gas with H_2_S	+/−	−	Acid/acid/gas
13	Acid/acid/gas	+/+	−	Alkaline/alkaline/gas
14	Acid/acid	+/−	+	Acid/acid/gas
15	Acid/acid/gas	+/+	−	Alkaline/acid/gas
16	Alkaline/acid/gas with H_2_S	+/+	−	Alkaline/acid/gas
17	Alkaline/acid/gas with H_2_S	−/−	−	Alkaline/acid/gas
18	Alkaline/acid/gas	+/+	−	Alkaline/alkaline/gas
19	Alkaline/acid/gas with H_2_S	+/+	−	Alkaline/alkaline/gas
20	Acid/acid/gas	+/+	−	Alkaline/alkaline/gas
21	Alkaline/acid/gas	−/−	−	Alkaline/acid/gas
22	Alkaline/acid/gas	−/−	−	Alkaline/acid/gas

+, positive result; −, negative result.

**Table 10:** Number of LAB from broiler chickens’ gastrointestinal tracts.

Organ	Period	Treatment 1	Treatment 2	Treatment 3	Treatment 4	Treatment 5
Duodenum	Day 21	15.56%	8.89%	11.11%	20.00%	8.89%
	Day 42	13.33%	15.56%	13.33%	13.33%	15.56%
Jejunum	Day 21	15.56%	11.11%	11.11%	17.78%	8.89%
	Day 42	13.33%	15.56%	13.33%	13.33%	15.56%
Ileum	Day 21	15.56%	11.11%	11.11%	17.78%	8.89%
	Day 42	13.33%	15.56%	13.33%	15.56%	15.56%
Cecum	Day 21	15.56%	11.11%	11.11%	17.78%	8.89%
	Day 42	13.33%	15.56%	13.33%	13.33%	15.56%
Colon	Day 21	15.56%	11.11%	11.11%	17.78%	6.67%
	Day 42	13.33%	15.56%	13.33%	13.33%	15.56%

**Table 11:** Biochemical analysis of LAB isolated from broilers that receive diet supplements with tamarind shell powder.

LAB	% Survival Rate in Acid (pH)		% Survival Rate in Condition			Capacity of Digestion			Catalase Test
	2.5	3.0	Bile Salt	Gastric Juice	Intestinal Fluid	Starch	Fat	Protein	
M1	0	22.45	0	0	0	+	+	−	−
M2	0	0	ND	ND	ND	ND	ND	ND	ND
M3	0	0	ND	ND	ND	ND	ND	ND	ND
M4	0	0	ND	ND	ND	ND	ND	ND	ND
M5	0	6.87	60.16	7.06	75.79	−	−	−	−
M6	21.42	0	68.75	5.98	28.68	+	+	+	+
M7	0	13.56	0	1.60	50.68	−	−	−	−
M8	0	0	ND	ND	ND	ND	ND	ND	ND
M9	0	0	ND	ND	ND	ND	ND	ND	ND
M10	0	0	ND	ND	ND	ND	ND	ND	ND
M11	0	0	ND	ND	ND	ND	ND	ND	ND
M12	0	15.64	17.30	13.89	3.06	+	+	+	−
M13	0	1.22	15.46	2.40	88.89	+	+	−	+
M14	0	48.06	15.79	97.22	2.37	+	+	−	+
M15	0	0	ND	ND	ND	ND	ND	ND	ND
M16	0	1.48	20.93	15.46	33.33	+	−	−	−

+, positive result; −, negative result; ND, not detected.

**Table 12:** Inhibition of pathogenic bacteria by selected LAB using cross-streak method.

LAB	Pathogen				
	*S. typhimurium*	*S. enteritidis*	*E. coli*	E20	S19
M1	+	+	+	+	+
M5	+	+	+	+	+
M6	+	+	+	−	+
M7	−	+	+	+	+
M12	+	+	+	+	+
M13	+	+	+	−	+
M14	−	+	+	−	+
M16	−	−	−	−	−

+, positive result; −, negative result.

## Data Availability

The original contributions presented in this study are included in the article/Supplementary Material. Further inquiries can be directed to the corresponding author.

## Supplementary Materials

The following supporting information can be downloaded at https://www.mdpi.com/article/10.3390/vetsci13060566/s1, Figure S1: Biochemical test and colony characteristics of pathogen strain S19; Figure S2: Biochemical test and colony characteristics of pathogen strain E20; Figure S3: Antibacterial activity of lactic acid bacteria strains M1 and M12 isolated from broilers.
