# Supplementary material for: Tamarindus indica Shells Powder Enhances Growth Performance, Hemato-Biochemical Parameters, Nutrient Utilization, and Gut Health in Broiler Chickens
Source: Vet Sci. 2026 Jun 8;13(6):566. doi: 10.3390/vetsci13060566 (PMC13308468; doi:10.3390/vetsci13060566)
Supplement: Supplementary file 1 [file vetsci-13-00566-s001.zip › vetsci-4312281-supplementary.pdf]

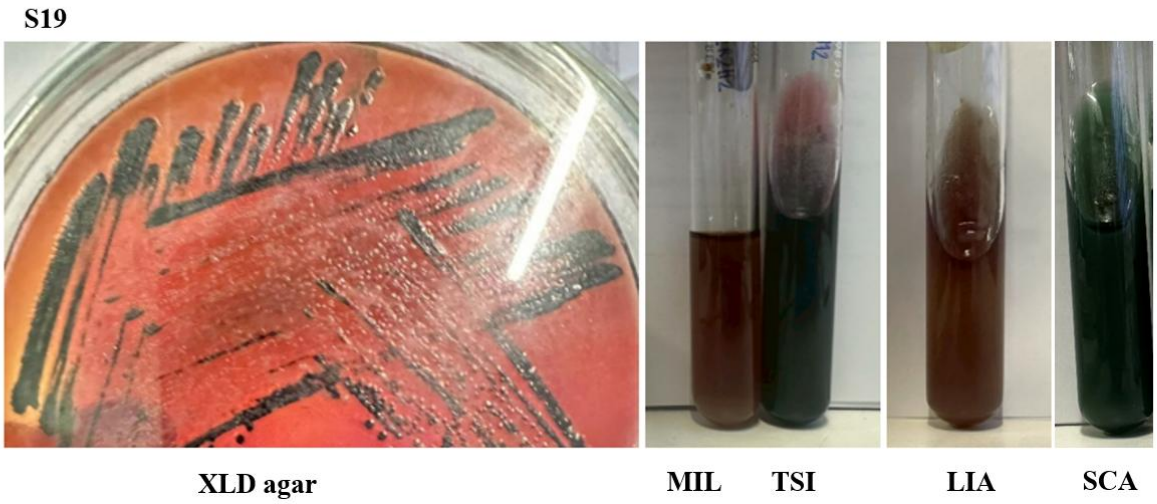

Figure S1. Biochemical test and colony characteristics of pathogen strain S19.

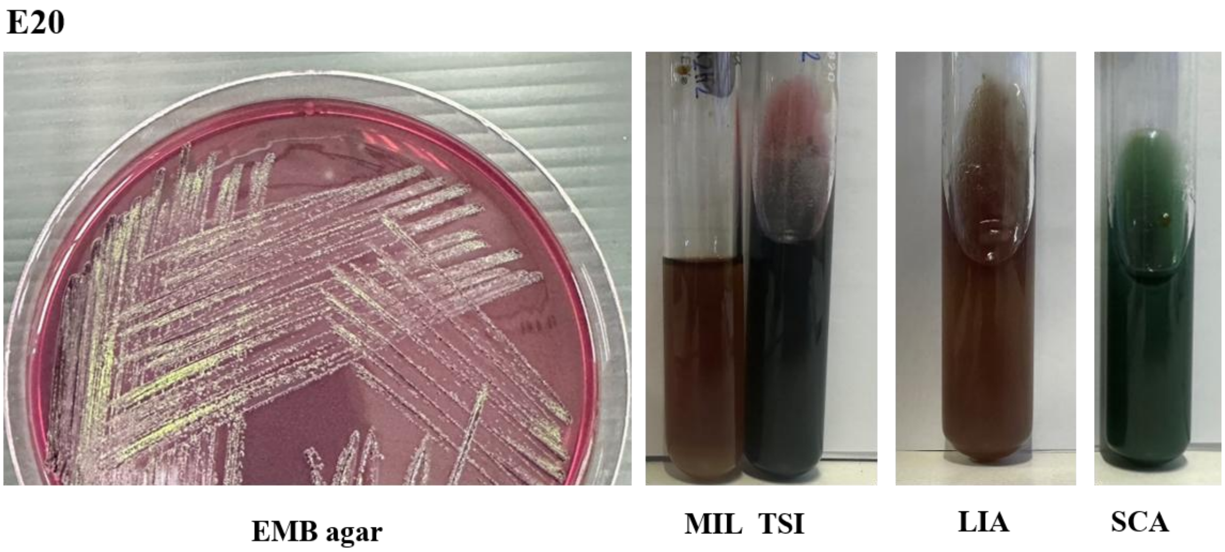

Figure S2. Biochemical test and colony characteristics of pathogen strain E20.

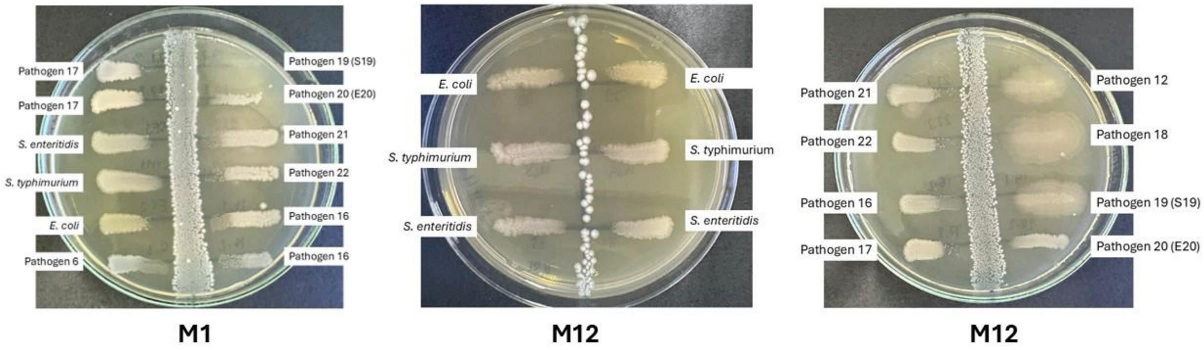

Figure S3. Antibacterial activity of lactic acid bacteria strains M1 and M12 isolated from broilers.
